# Second-Hand Cigarette Smoke Impairs Bacterial Phagocytosis in Macrophages by Modulating CFTR Dependent Lipid-Rafts

**DOI:** 10.1371/journal.pone.0121200

**Published:** 2015-03-20

**Authors:** Inzer Ni, Changhoon Ji, Neeraj Vij

**Affiliations:** 1 Department of Pediatric Respiratory Science, The Johns Hopkins University School of Medicine, Baltimore, Maryland, United States of America; 2 Department of Biomedical Engineering, The Johns Hopkins University School of Medicine, Baltimore, Maryland, United States of America; 3 Department of Foundational Sciences, College of Medicine, Central Michigan University, Mount Pleasant, Michigan, United States of America; National Jewish Health, UNITED STATES

## Abstract

**Introduction:**

First/Second-hand cigarette-smoke (FHS/SHS) exposure weakens immune defenses inducing chronic obstructive pulmonary disease (COPD) but the underlying mechanisms are not fully understood. Hence, we evaluated if SHS induced changes in membrane/lipid-raft (m-/r)-CFTR (cystic fibrosis transmembrane conductance regulator) expression/activity is a potential mechanism for impaired bacterial phagocytosis in COPD.

**Methods:**

RAW264.7 murine macrophages were exposed to freshly prepared CS-extract (CSE) containing culture media and/or *Pseudomonas-aeruginosa-PA01*-GFP for phagocytosis (fluorescence-microscopy), bacterial survival (colony-forming-units-CFU), and immunoblotting assays. The CFTR-expression/activity and lipid-rafts were modulated by transient-transfection or inhibitors/inducers. Next, mice were exposed to acute/sub-chronic-SHS or room-air (5-days/3-weeks) and infected with *PA01*-GFP, followed by quantification of bacterial survival by CFU-assay.

**Results:**

We investigated the effect of CSE treatment on RAW264.7 cells infected by *PA01*-GFP and observed that CSE treatment significantly (p<0.01) inhibits *PA01*-GFP phagocytosis as compared to the controls. We also verified this in murine model, exposed to acute/sub-chronic-SHS and found significant (p<0.05, p<0.02) increase in bacterial survival in the SHS-exposed lungs as compared to the room-air controls. Next, we examined the effect of impaired CFTR ion-channel-activity on *PA01*-GFP infection of RAW264.7 cells using CFTR172-inhibitor and found no significant change in phagocytosis. We also similarly evaluated the effect of a CFTR corrector-potentiator compound, VRT-532, and observed no significant rescue of CSE impaired *PA01*-GFP phagocytosis although it significantly (p<0.05) decreases CSE induced bacterial survival. Moreover, induction of CFTR expression in macrophages significantly (p<0.03) improves CSE impaired *PA01*-GFP phagocytosis as compared to the control. Next, we verified the link between m-/r-CFTR expression and phagocytosis using methyl-β-cyclodextran (CD), as it is known to deplete CFTR from membrane lipid-rafts. We observed that CD treatment significantly (p<0.01) inhibits bacterial phagocytosis in RAW264.7 cells and adding CSE further impairs phagocytosis suggesting synergistic effect on CFTR dependent lipid-rafts.

**Conclusion:**

Our data suggest that SHS impairs bacterial phagocytosis by modulating CFTR dependent lipid-rafts.

## Introduction

COPD consists of two main conditions, chronic bronchitis and emphysema [1,2,3], and is projected to be the third most common cause of death globally by 2020 [4,5]. First- and second- hand cigarette smoke (FHS/SHS) are among the most common causes of chronic obstructive pulmonary disease (COPD) in the United States (US), which leads to lung damage and airspace enlargement or emphysema [5,6,7]. Cigarette smoke (CS) contains over 4,000 different chemical compounds and is known to induce inflammatory-oxidative stress in the lungs initiating COPD-emphysema [8,9,10,11]. Furthermore, chronic exacerbations (viral and bacterial infections) in COPD subjects initiate a chronic inflammatory lung disease [2,12,13] due to weakened immune defenses. Although COPD subjects have five- to ten- times more alveolar macrophages as compared to the healthy individuals [14,15] but these cannot combat increased bacterial infection and colonization suggesting impaired clearance and phagocytosis mechanisms [16,17]. In contrast in healthy individuals, alveolar macrophages are a critical component of the lung’s innate immune defense as they can clear off infection by mechanisms such as phagocytosis [18,19]. Specifically, CS exposure impairs the phagocytosis function of alveolar macrophages, which can deteriorate further with age and/or recurring infection(s) leading to severe COPD-emphysema pathogenesis and lung function decline [5,14,20].

In fact, previous studies have suggested a correlation between CS induced COPD (CS-COPD) and increased risk of bacterial infection due to impaired phagocytosis by neutrophils or alveolar macrophages [10,18,21,22,23,24,25,26]. Moreover, murine studies have shown that smoke exposure delays *P*. *aeruginosa* clearance from the lungs and induces inflammatory cytokine secretion resulting in chronic lung disease [17,21,26]. We selected to evaluate here the specific mechanism for *P*. *aeruginosa* exacerbation as it is a common pathogen involved in pathogenesis of chronic obstructive lung diseases such as cystic fibrosis (CF) and COPD [17,24,27]. Notably, both “*mutations*”- (CF) and “*acquired*”- CFTR (Cystic Fibrosis Transmembrane Conductance Regulator) dysfunction (COPD) are known to be associated with pathogenesis of chronic obstructive lung disease [6,12,28,29]. In addition, *P*. *aeruginosa* is known to adapt to the human airways of both CF and COPD subjects by secreting an extracellular matrix proteins that form a biofilm involved in pathogenesis of chronic lung disease [13,27,30,31,32]. Furthermore, these chronic bacterial exacerbations are the major cause of mortality in CF/COPD subjects [22,24].

Smoke exposure has been shown to deplete CFTR from cell membrane (m-) and lipid-rafts (r-), while increasing its internalization, and endoplasmic reticulum (ER) accumulation as a result of impaired peripheral- and ER- associated degradation [7,12,28,33]. Moreover, recent studies verify that dysfunctional CFTR (mutation/acquired) impairs bacterial clearance by macrophages [18,34,35] that increase susceptibility to bacterial infections [12,36,37,38,39,40]. The CFTR deficiency has also been linked to defective reactive oxidative species (ROS) and acid sphingomyelinase (ASMase) activation, which are required for bacterial killing by macrophages [41,42]. Despite the intriguing evidence on the pathogenic role of smoke exposure in increasing the risk of bacterial infection [5,10,15,18], the underlying mechanism(s) by which SHS exposure impairs CFTR dependent bacterial phagocytosis in macrophages is not fully understood.

Hence, we evaluated if SHS induced m-/r- CFTR dysfunction in macrophages is a critical mechanism for impaired bacterial phagocytosis in COPD. We first verified the pathogenic role of SHS in diminishing bacterial phagocytosis that can initiate chronic obstructive lung disease. We also found that SHS exposure can modulate CFTR dependent lipid-rafts as a potential mechanism for impaired bacterial phagocytosis.

## Materials and Methods

### Reagents and treatments

**Fig 1:**
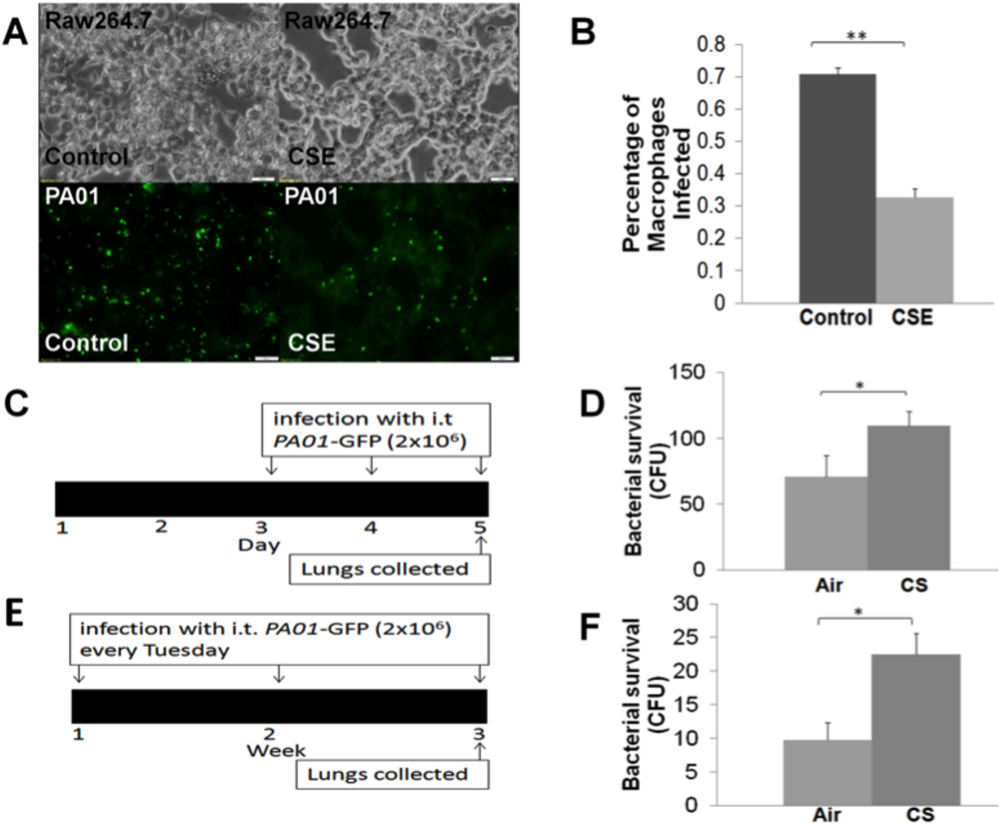
Secondhand cigarette smoke exposure impairs bacterial phagocytosis and improves survival. (A) RAW264.7 cells were seeded on a 24-well plate and infected with *PA01*-GFP (multiplicity of infection, MOI 10) and/or treated with cigarette smoke extract (CSE; 10%) for 150 min as a secondhand cigarette smoke (SHS) exposure model, followed by microscopy. Representative bright field (top) and fluorescent images (bottom) are shown (magnification 40X, n = 4, white bar = 20μm). (B) CSE treatment as above, significantly (**p<0.01) impairs bacterial phagocytosis in RAW264.7 cells. (C) C57BL6 mice were exposed to either room-air (control) or SHS for 5 days. Mice were infected with intra-tracheal (i.t.) *PA01*-GFP (2x10^6^ at the indicated time points on the scale), followed by shredding of lungs for bacterial survival assay. (D) Acute-SHS exposed mice show significantly (*p<0.05) higher bacterial load (colony forming units, CFU) in the shredded lungs (low speed sonication; 1 pulse 10 sec) as compared to the room-air controls (n = 4, left: bar graph, right: scatter plot of same data). (E) The C57BL6 were exposed to either room-air (control) or SHS for 3 weeks (5 days/week) and were infected with intra-tracheal (i.t.) *PA01*-GFP (2x10^6^ at the indicated time points on the scale). Lungs were shredded and assessed for bacterial load by quantifying colony-forming units (CFU) at the end of the 3-week period. (F) The lungs of sub-chronic- SHS exposed mice show significantly (*p<0.02, > 2 fold) greater bacterial survival (n = 4, left: bar graph, right: scatter plot of same data) as compared to the room-air controls.

The murine macrophage cell line, RAW264.7 was cultured at 37°C with 5% CO_2_ in Dulbecco's Modified Eagle Medium: Nutrient Mixture F-12 (DMEM-F-12; Corning Cellgro, Manassas, VA) media. The media was supplemented with 10% Fetal Bovine Serum (FBS; Corning Cellgro) and 1% Penicillin, Streptomycin, Amphotericin B (PSA; Corning Cellgro). For *in-vitro* second hand smoke (SHS) exposure experiments, cigarette smoke extract (CSE) was directly collected in culture media and used at a concentration of 10% for 150 minutes (min) exposure as described below. To inhibit cystic fibrosis transmembrane conductance regulator (CFTR) channel activity in RAW264.7 cells, CFTR(inh)-172 (Sigma-Aldrich, St. Louis, MO) was used at a concentration of 10μM for overnight treatments. Similarly overnight treatment with flavonoid, Rutin Hydrate (10μM; Sigma-Aldrich) or Quercetin (10μM; Sigma) was used to modulate CFTR channel activity. To test the effect of increased CFTR expression/activity on phagocytosis, RAW264.7 cells were treated overnight with VRT-532 (10μM; CF Foundation, Bethesda, MD) [43,44]. While effect of CFTR-lipid-raft disruption was evaluated using the CFTR/cholesterol depleting reagent methyl-β-cyclodextran (CD; Sigma) at a concentration of 5mM for 16 hours (hrs). RAW264.7 cells were also transiently transfected for 48 hrs with the empty vector pcDNA3.1 or pcDNA3.1-WTCFTR plasmid using the Lipofectamine 2000 reagent (Invitrogen, Carlsbad, CA) following the manufacturer’s instructions. For infection experiments, *Pseudomonas aeruginosa* (*P*. *aeruginosa*) strain *PA01*-GFP was cultured overnight in Luria Bertani (LB; Thermo Fisher Scientific, Waltham, MA) broth supplemented with GFP selection antibiotic marker (1% carbenicillin; Sigma) at 37°C in a shaking incubator at 250 rpm. For in vitro experiments, *PA01*-GFP was directly added to DMEM-F12 media at indicated MOIs (multiplicity of infection) as described below while murine studies used intratracheal instillation (i.t.) of 2x10^6^ *PA01*-GFP in 25μL phosphate buffered saline (PBS; Corning Cellgro) at indicated time points (Fig. 1C and E).

### In vitro second hand smoke exposure (SHS) model

Research grade cigarettes (3R4F, 0.73 mg nicotine/cigarette) were purchased from the Tobacco Research Institute (University of Kentucky, Lexington, KY) and were used for SHS exposure. Briefly, side stream smoke (bubbling, instead of direct first-hand smoke exposure) from five-research grade cigarettes (no filters, to provide SHS similar to environmental exposure) was bubbled into 10mL of DMEM-F-12 media using a vacuum flask [23,45]. SHS containing media was diluted to standardize the optical density (OD) at 0.74 +/-0.06 (~pH 7.4) using VersaMax absorbance microplate reader (340nm, Molecular Devices, Sunnyvale, CA), to obtain 10%-CSE concentration [46]. This working concentration of 10% CSE was used as an *in vitro* (SHS) exposure model by adding CSE containing culture media at indicated time points [47].

### Immunoblotting

Total protein extract was isolated using 1X Radio-Immunoprecipitation Assay buffer (RIPA; Sigma) supplemented with ethylene-di-amine-tetra-acetic-acid (1%; EDTA, Sigma) and protease inhibitor cocktail (1%; Sigma). Total protein concentration was quantified using Protein Assay Reagent (Bio-Rad, Hercules, CA) and VersaMax absorbance microplate reader (595nm, Molecular Devices). The 80μg of total protein was loaded in each well. Changes in protein expression were quantified by immunoblotting for CFTR (596-ab; CF Foundation), NF-κB (Santa Cruz Biotechnology, Santa Cruz, CA), or β-actin (Sigma) antibodies. The secondary antibodies were anti-mouse (Amersham, Amersham, UK) and anti-rabbit (Amersham).

### Fluorescence microscopy for functional quantification of phagocytosis

RAW264.7 cells were seeded onto 24 well plates and treated overnight with CFTR(inh)-172 (10μM), rutin hydrate (10μM), quercetin (10μM), VRT-532 (10μM) or CD (5mM), or transiently transfected for 48 hours with control pcDNA3.1 vector or pcDNA3.1-WTCFTR plasmid construct. These cells were exposed to SHS (10%-CSE) for 150 min and concurrently infected with *PA01*-GFP at multiplicity of infection (MOI) 10 before fluorescent images were taken using Zeiss Inverted Microscope equipped with Olympus Camera and CellSens software. Images were taken at room temperature with air as the imaging medium. GFP and bright field images of representative areas were taken at 40X magnification with image capture set at LD Plan-Neo Fluor (40X/0.6X Phz Korr) and with 1.6X optivar. Phagocytosis analysis was performed by counting *PA01*-GFP infected (fluorescent) and total (bright field) macrophages in images captured from the same field. The percentage of macrophages that were infected was calculated by dividing the number of GFP positive macrophages by total number of macrophages.

### Bacterial survival assay

RAW264.7 cells were seeded onto 24-well plates and treated overnight with CFTR(inh)-172 (10μM), rutin hydrate (10μM), quercetin (10μM), VRT-532 (10μM), or CD (5mM) as indicated. Alternatively, cells were transiently transfected for 48 hours with control pcDNA3.1 vector or pcDNA3.1-WTCFTR plasmid. These cells were then infected with *PA01*-GFP at MOI 10 for 150min and concurrently exposed to SHS (10% CSE) as indicated. The media was collected from these cultures and serially diluted in 100μL volumes of PBS that was spread on 2% LB agar plates supplemented with 1% carbenicillin (selection antibiotic marker). The plates were incubated at 37°C overnight and bacteria colony forming unit (CFU) counts were used to quantify bacterial survival.

### Secondhand cigarette smoke (SHS) exposure and P. aeruginosa infection in murine model

All animal experiments were carried out in accordance with the Johns Hopkins University Animal Care and Use Committee (JHU-ACUC) approved protocol. C57BL6 mice were housed in a temperature/humidity controlled environment and exposed to room-air or non-filtered side stream SHS (instead of direct airway exposure to filtered first-hand smoke) was puffed by TE-2 cigarette smoking machine (Teague Enterprises, Davis, CA) into the exposure chamber using research grade cigarettes (3R4F, 0.73 mg nicotine/cigarette) purchased from the Tobacco Research Institute (University of Kentucky, Lexington, KY) as described recently [6,12]. The smoke exposure time for these experiments was five hours for 5 days for the acute SHS exposure experiment or 3 weeks (5 days/week) for the sub-chronic SHS exposure experiment. The control groups of mice were exposed to room-air for either 5 days (acute) or 3 weeks (sub-chronic). Both SHS and room-air exposed mice were also infected with intra-tracheal (i.t.) *PA01*-GFP (2x10^6^/day) on days 3–5 for the acute SHS experiment or once every week for the sub-chronic SHS experiment. Mice were euthanized following ACUC approved protocols, and lungs were collected and gently shredded in 1mL PBS using low speed sonication (1 pulse 10 sec). These lung extracts were then assessed for bacterial load at the end of the five day or three week period by spreading 100μL of the lung lysate onto 2% agar plates supplemented with 1% carbenicillin. CFU counts were obtained after overnight incubation as described above.

### Statistical analysis

Data is presented as the mean ± standard error of the mean (SEM) and variations in data between the different groups were tested using Student t-test. Differences were considered significant if p values were <0.05. Densitometry analysis of immunoblotting results was done using Image J software (NIH, Bethesda, MD) and fluorescence microscopy analysis was performed using Adobe Photoshop CS5 (Adobe Systems, San Jose, CA).

## Results

### Secondhand cigarette smoke exposure inhibits bacterial phagocytosis and increases survival

We first verified if secondhand cigarette smoke (SHS) exposure impairs bacterial phagocytosis in macrophages by treating RAW 264.7 cells with 10% cigarette smoke extract (CSE) and infecting them with *Pseudomonas aeruginosa* (*P*. *aeruginosa*) strain *PA01*-GFP (multiplicity of infection, MOI 10). Fluorescent microscopy images were captured and analyzed using Zeiss Inverted Microscope equipped with camera and analysis software. We found that 10% CSE treatment significantly (p<0.01) impairs bacterial phagocytosis in RAW264.7 macrophages as compared to controls (Fig. 1A, B). In a parallel study, we used a murine model where C57BL6 mice were exposed to either room-air or acute/sub-chronic- SHS. These mice were also infected with intra-tracheal (i.t.) *PA01*-GFP (2x10^6^) at the indicated time points (see scale, Fig. 1C and E) to determine impact of SHS exposure on bacterial survival. The alveolar bacterial CFU counts were quantified for room-air and SHS treated groups as described above to quantify the total amount of surviving *PA01*-GFP in the lungs. The acute-SHS exposed murine lungs had significantly (p<0.05) higher bacterial load as compared to lungs from room-air exposed controls (Fig. 1D). We observed that increasing SHS exposure time in sub-chronic model leads to even more significant (p<0.02, >2 fold change) increase in alveolar bacterial load as compared to room-air controls (Fig. 1F) although basal bacterial load in these mice was lower as same number of bacteria (as acute-SHS exposure) was intra-tracheally instilled over a longer time frame. These findings confirm the detrimental effect of SHS exposure seen in the *in vitro* model (Fig. 1A-B). Our data suggest that SHS impairs bacterial phagocytosis and may improve their survival in RAW264.7 macrophages. Overall, we observed that side stream SHS exposure increases bacterial burden (Fig. 1D and F) in the murine lungs.

### Modulating CFTR channel activity does not restore SHS impaired bacterial phagocytosis

**Fig 2:**
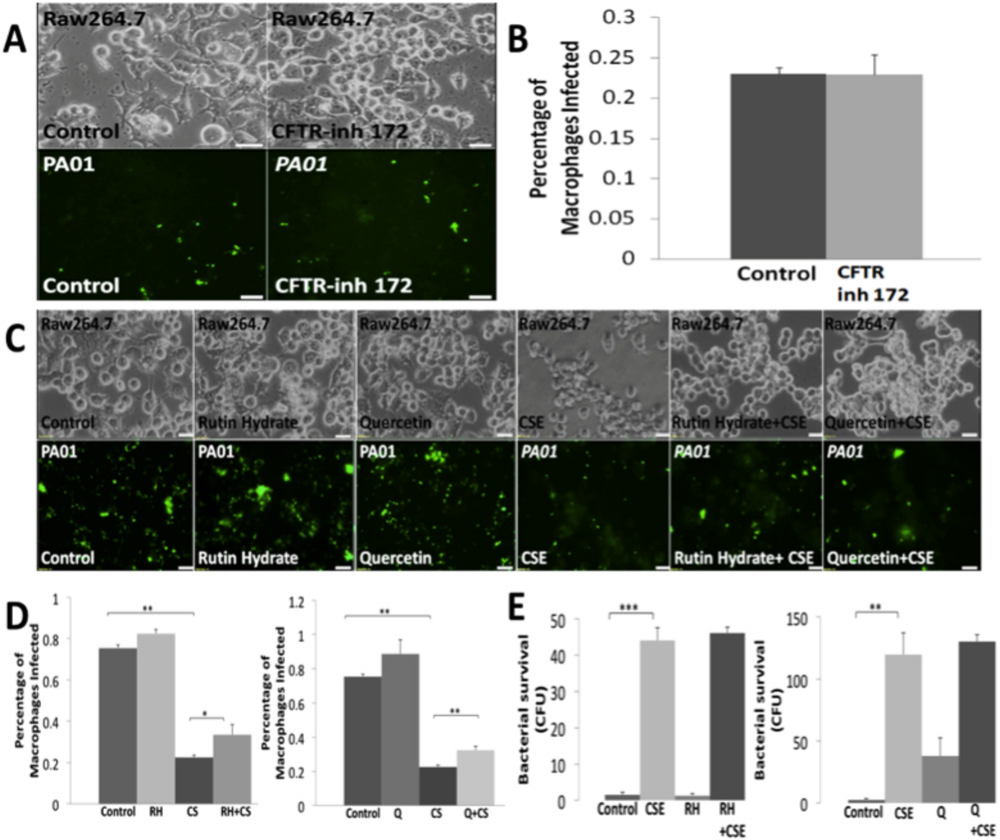
CFTR ion channel activity modulators cannot restore SHS impaired bacterial phagocytosis and limit survival. (A) RAW264.7 cells were seeded on a 24-well plate and treated overnight with CFTR(inh)-172 (10 μM). In addition, cells were infected with *PA01*-GFP (MOI 10) for 150min followed by microscopy. Representative bright field (top) and fluorescent images (bottom) are shown (magnification 40X, n = 6, white bar = 20μm). (B) Next we quantified (from 2A) the percentage of macrophages that were infected with *PA01*-GFP and found that inhibiting CFTR ion channel activity does not impair bacterial phagocytosis. (C) RAW264.7 cells were seeded on a 24-well plate and treated overnight with the flavonoids, Rutin Hydrate (RH, 10μM) or Quercetin (Q, 10μM). Cells were also infected with *PA01*-GFP (MOI 10) and/or treated with cigarette smoke extract (CSE; 10%; SHS model) for 150 min followed by microscopy. The representative bright field (top) and fluorescent images (bottom) are shown (magnification 40X, n = 6, white bar = 20μm). (D) The quantification (from 2C) of bacterial phagocytosis shows significant (*p<0.05, **p<0.01) increase in Q or RH and CSE treated groups as compared to the CSE treated control group. But this change is not sufficient to restore SHS impaired bacterial phagocytosis to the levels seen in the control group. (E) In a separate experiment (similar to one described in 2C), media (100μl) was collected and spread on 2% LB agar plates. The plates were incubated overnight at 37°C and colony forming unit (CFU) counts were used to quantify bacterial survival. CSE treatment significantly (**p<0.01) improves bacterial survival, but RH or Q treatment of CSE group does not significantly affect bacterial counts.

We and others recently found that CS exposure decreases cystic fibrosis transmembrane conductance regulator (CFTR) expression and function [12,33,48]. Moreover, CFTR has been linked to impaired bacterial clearance in macrophages [18,34,41,42,49]. Therefore we first tested if CFTR ion channel activity plays a role in macrophage mediated immune defenses against bacteria. Hence, RAW264.7 cells were treated with the flavonoids quercetin, a known ion channel activator, and its glycoside, rutin hydrate. Although quercetin is known to activate CFTR ion channel at low doses, it acts as an inhibitor at higher doses [50,51]. Thus, these flavonoids were used at a lower concentration to investigate the impact of CFTR ion channel activation on bacterial phagocytosis. RAW264.7 cells were treated with flavonoids (10μM of rutin hydrate or quercetin) followed by infection with *PA01*-GFP (MOI 10) and/or 10% CSE treatment. The microscopic analysis of *PA01*-GFP shows a significant increase in phagocytosis in the quercetin + CSE (p<0.05) and rutin hydrate + CSE (p<0.01) treated groups as compared to the CSE, but this change is not sufficient to restore or revert SHS (CSE) impaired bacterial phagocytosis to the levels seen in the control group (Fig. 2C-D). Bacterial survival was also significantly (p<0.01) higher in all CSE treated groups as compared to controls. Moreover, the use of overnight rutin hydrate or quercetin treatment was unable to inhibit SHS induced bacterial burden (survival, Fig. 2E). We further investigated the effect of CFTR ion channel inhibition on bacterial phagocytosis by treating RAW264.7 cells with CFTR(inh)-172 (10 μM) inhibitor [52]. The percentage of macrophages that were infected with *PA01*-GFP did not vary between CFTR(inh)-172 inhibitor treated group as compared to the control, which indicate that inhibiting CFTR ion channel activity does not impair bacterial phagocytosis (Fig. 2A-B). Data suggest that CFTR ion channel activity may not be critical for bacterial phagocytosis, as modulation of CFTR channel activity could not rescue SHS impaired bacterial phagocytosis to the basal levels (Fig. 2).

### CFTR expression rescues SHS impaired phagocytosis and limits survival

**Fig 3:**
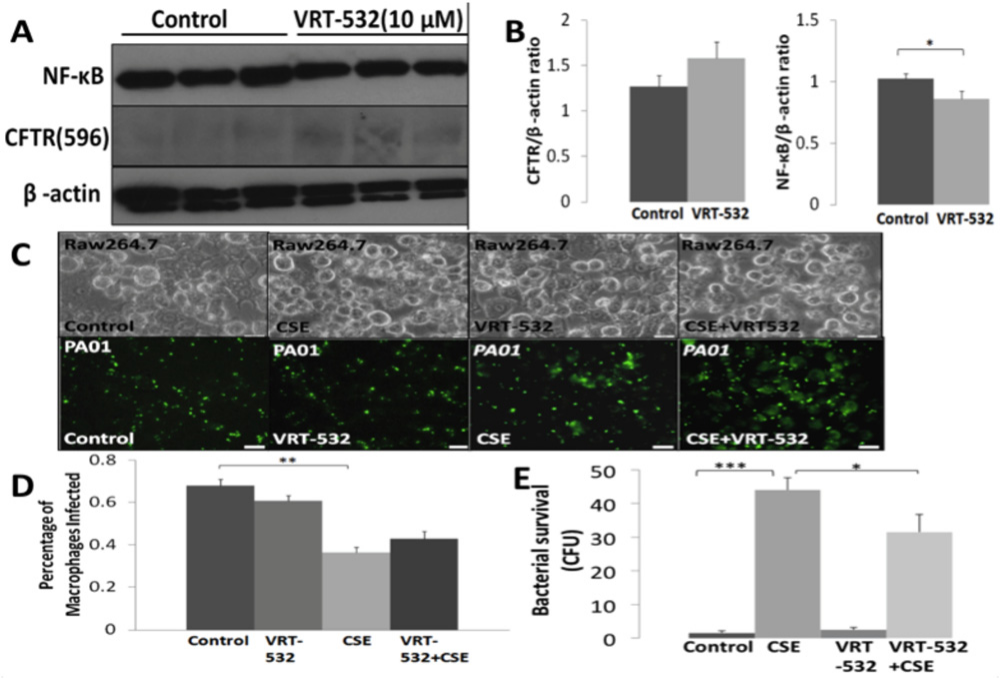
Modulating CFTR expression may limit SHS induced bacterial survival. (A) Immunoblotting of total protein extracts from RAW264.7 cells treated with VRT-532 (10μM; overnight, a known CFTR corrector and potentiator) or untreated group (control), show slightly higher protein levels of CFTR and reduced NF-κB levels in the treatment group as compared to the controls. β-actin was used as a loading control (n = 3). (B) CFTR and NF- κB protein expression (in 3A) was normalized to β—actin using an Image-J software. The densitometry analysis verifies that VRT-532 treatment can decrease NF-κB protein expression (*p<0.05) as compared to untreated control. (C) RAW264.7 cells were seeded on a 24-well plate and treated overnight with VRT-532 (10μM). Next, these cells were infected with *PA01*-GFP (MOI 10) and/or treated with cigarette smoke extract (CSE; 10%; SHS model) for 150mins. Representative bright field (top) and fluorescent microscopy images (bottom) are shown (magnification 40X, n = 4, white bar = 20μm). (D) CSE treatment (in 3C) significantly (**p<0.01) inhibits bacterial phagocytosis, while VRT-532 is unable to restore SHS impaired phagocytosis. (E) In a separate experiment (as described in 3C), media (100μl) was collected, spread on agar plates, and then incubated overnight at 37°C. CFU counts of the extracellular bacteria (media) indicates that VRT-532 treatment can significantly (*p<0.05) decrease SHS induced bacterial survival. Note, this experiment was done in parallel with Fig. 2E (left panel, Rutin Hydrate), hence control and CSE samples used are common.

**Fig 4:**
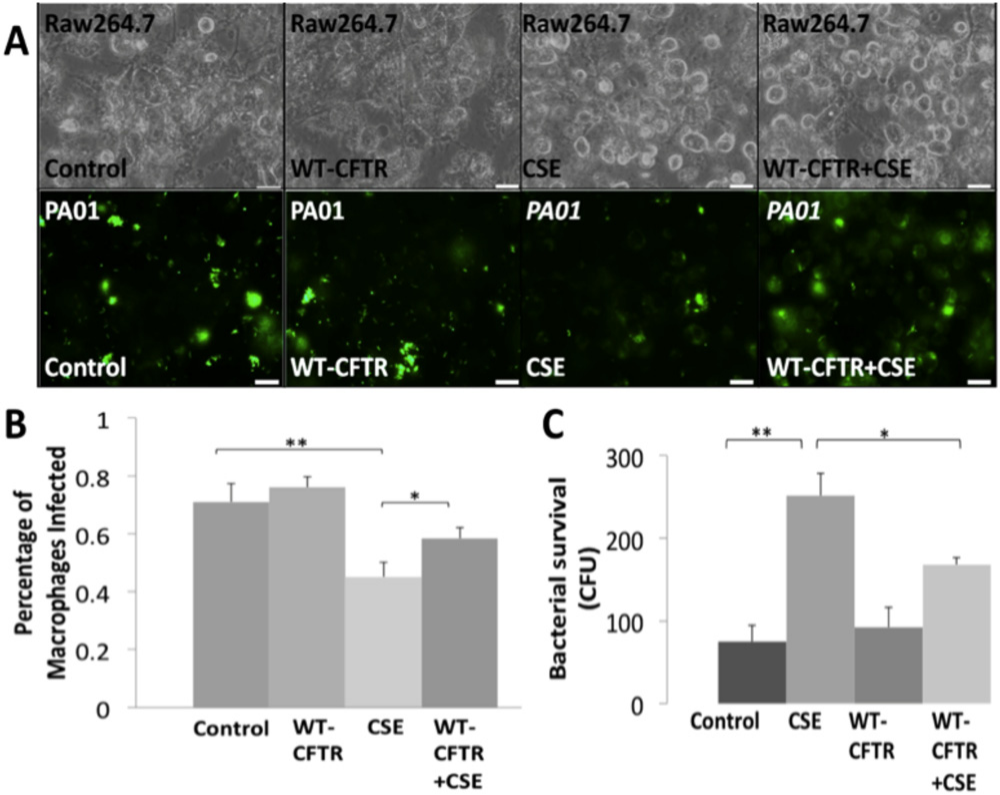
Transient transfection with WT-CFTR rescues SHS impaired bacterial phagocytosis and limits survival. (A) RAW264.7 cells were seeded on a 24-well plate and transiently transfected (using Lipofectamine) with pcDNA3.1 control vector or pcDNA3.1-WTCFTR for 48 hours. Cells were infected with *PA01*-GFP (MOI 10) and/or treated with cigarette smoke extract (CSE; 10%; SHS model) for 150 min before microscopy. Representative bright field (top) and fluorescent images (bottom) are shown (magnification 40X, n = 4, white bar = 20μm). (B) Quantification of bacterial phagocytosis (from 4A) shows that transfection with WT-CFTR significantly (*p<0.03) rescues SHS impaired phagocytosis. (C) RAW264.7 cells were treated (as described in 4A) and media (100 μL) was collected and spread on 2% LB agar plates. The plates were incubated overnight at 37°C followed by quantification of bacterial survival by colony forming unit (CFU) counts. Transient transfection with WT-CFTR significantly (*p<0.02) limits bacterial survival in the CSE/SHS treated group, but bacterial survival remains higher than the untreated controls suggesting higher CFTR expression may be needed to limit bacterial burden.

We investigated if induction of CFTR expression and activity can improve CSE impaired bacterial phagocytosis in macrophages. For these experiments, RAW264.7 cells were treated with VRT-532, a known CFTR corrector and potentiator. We first quantified changes in CFTR and NF-κB protein levels by immunoblotting of total protein extracts and found that VRT-532 treatment slightly induces CFTR protein levels while significantly (p<0.05) inhibiting NF-κB (Fig. 3A-B). Next, we treated RAW264.7 cells with VRT-532 (10μM; overnight) followed by *PA01*-GFP (MOI 10; 150min) infection and 10% CSE treatment. As in previous experiments, we found that 10% CSE treatment significantly (p<0.01) inhibits bacterial phagocytosis while VRT-532 slightly induces bacterial phagocytosis. The data suggest that CFTR induction by VRT-532 is not significant or potent to completely rescue CSE impaired bacterial phagocytosis (Fig. 3C-D). Although, CFU counts of the extracellular bacteria demonstrate that VRT-532 treatment can significantly (p<0.05) decrease CSE induced bacterial burden (survival, Fig. 3E). This can be explained, as VRT-532 treatment minimally increases CFTR expression in RAW264.7 cells but this minimal activation may not be potent enough to restore SHS impaired bacterial phagocytosis to the basal levels. We further investigated the role of increased CFTR expression on bacterial phagocytosis by transfecting RAW264.7 cells with pcDNA3.1-WT-CFTR, as VRT-532 was not potent in significantly inducing CFTR expression. We found that inducing WT-CFTR expression significantly (p<0.03) rescues CSE impaired bacterial phagocytosis as compared to pcDNA3.1 vector control group (Fig. 4A-B). This induction of SHS impaired bacterial phagocytosis by CFTR expression suggests the role of CFTR dependent lipid-rafts. Moreover, transient transfection with WT-CFTR is able to significantly (p<0.02) limit bacterial survival in the CSE treated group but it does not restore the basal levels (Fig. 4C). We anticipate that further induction of CFTR dependent lipid-rafts can fully restore SHS impaired bacterial phagocytosis to combat chronic infection.

### Lipid-raft disruption inhibits bacterial phagocytosis

**Fig 5:**
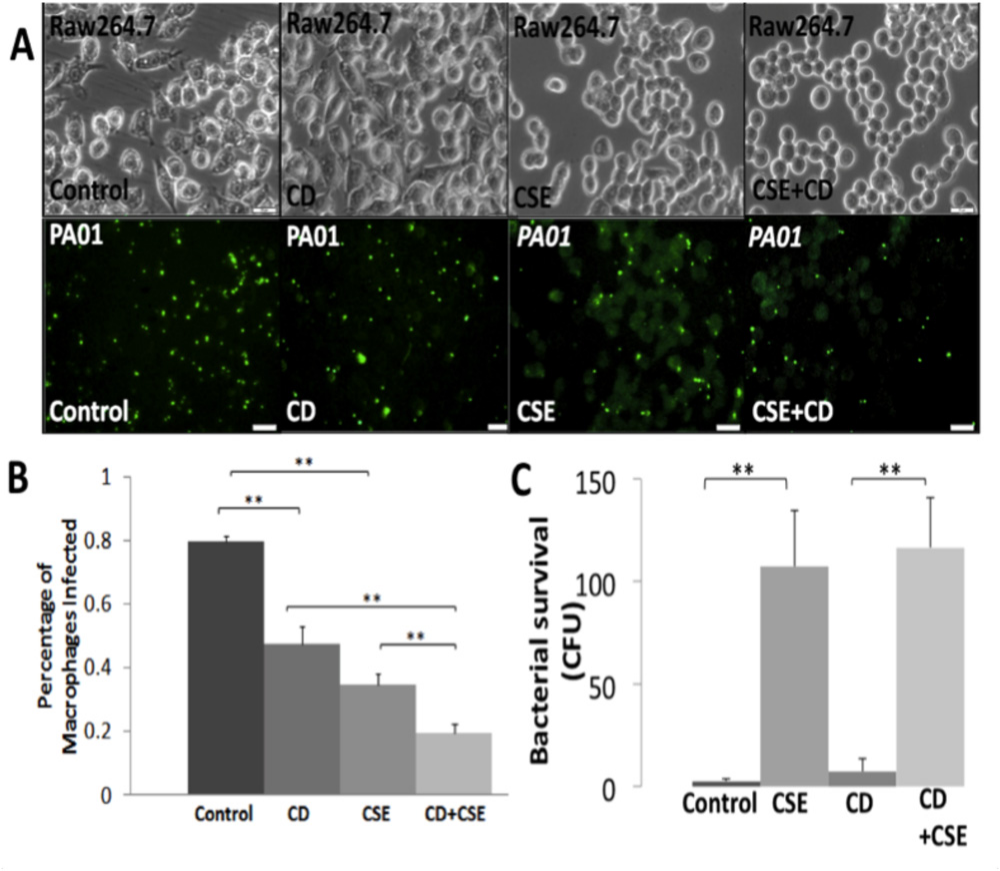
Lipid-raft disruption impairs bacterial phagocytosis. (A) RAW264.7 cells were seeded on a 24-well plate and treated overnight with methyl-β-cyclodextran (CD; 5mM), followed by *PA01*-GFP (MOI 10) and/or cigarette smoke extract (CSE; 10%; SHS model) treatment for 150 mins. Representative bright field (top) and fluorescent microscopy images (bottom) images are shown (magnification 40X, n = 4, white bar = 20μm). (B) Overnight CD treatment (in 5A) significantly (**p<0.01) impairs bacterial phagocytosis as compared to untreated controls. In addition, CD treatment further inhibits SHS impaired bacterial phagocytosis suggesting the role of CFTR dependent lipid-rafts in this process. (C) RAW264.7 cells were treated (as described in 5A), and media (100μl) was collected and spread on 2% LB agar plates, followed by overnight incubation at 37°C. CSE treatment significantly (**p<0.01) induces bacteria survival while CD treatment slightly elevates CSE induced bacterial survival.

Macrophages (RAW264.7) were treated with methyl-β-cyclodextran (CD), a known lipid-raft inhibitor that depletes CFTR and cholesterol from membrane microdomains, to examine the effect of CFTR-raft disruption on bacterial phagocytosis. Briefly, cells were treated overnight with CD at 5mM followed by *PA01*-GFP (MOI 10) infection and 10% CSE treatment. We found that CD treatment significantly (p<0.01) impairs bacterial phagocytosis as compared to untreated controls suggesting the role of CFTR dependent lipid-rafts in this process. In addition, CSE treatment together with CD further decreases (~1.77 fold) bacterial phagocytosis suggesting a synergistic inhibition of CFTR dependent lipid-rafts as a potential mechanism to impair phagocytosis (Fig. 5A-B). Similar to previous experiments, we again found that CSE treatment significantly (p<0.01) elevates bacterial survival in RAW264.7 cells but addition of CD cannot further induce the effect of SHS on bacterial survival. We anticipate that CD may be inhibiting bacterial growth directly [53] leading to the decreased extracellular bacterial counts contrary to the expectation.

## Discussion

First- and second- hand cigarette smoke (FHS/SHS) induced chronic obstructive pulmonary disease (COPD) has been linked to increased risk of bacterial infection as CS can impair immune defense functions of neutrophils and alveolar macrophages (18,22,23). In this study, we found that SHS exposure significantly inhibits bacterial (*P*. *aeruginosa PA01*-GFP) phagocytosis while improving the survival suggesting this as a mechanism for smoke induced exacerbations in COPD subjects (Fig. 1A-B). We also verified that acute- and sub-chronic- SHS exposure of C57BL6 mice induces alveolar bacterial survival (Fig. 1C-F). This supports previous studies in CS-murine model (chronic smoke) that observed suppression of antibacterial (*P*. *aeruginosa*) immune defenses by cigarette smoke [17,21,26]. Furthermore, the alveolar macrophages of COPD patients have impaired bacterial (*Haemophillus influenza*, *Escherichia coli*, *and Streptococcus pneumonia*) phagocytosis [5,54], but the specific mechanism is not known. We found that SHS impaired phagocytosis improves *P*. *aeruginosa* survival in the airways that can induce pathogenesis of chronic obstructive lung disease.

Moreover, we anticipated that SHS impaired bacterial phagocytosis involves cystic fibrosis transmembrane conductance regulator (CFTR) as it is known to modulate *P*. *aeruginosa* phagocytosis while SHS can deplete CFTR from membrane/lipid-rafts (m-/r-) [12,38,55]. CFTR is conventionally believed to be an ion channel that is expressed in both human and mouse alveolar macrophages but its function and mechanism(s) in inflammatory cells are not well-studied [34,42,56,57]. Recent studies have shown that CS can suppress CFTR expression and activity [7,12,33,58,59]. As discussed above, CS can deplete m-/r-CFTR expression and activity [6,7,33,48,58] that can impair bacterial clearance [18,41]. Hence, in this study, we first investigated the role of CFTR ion channel activity in bacterial phagocytosis using the RAW264.7 cell line, which endogenously expresses CFTR [60,61]. We tested the flavonoids quercetin and rutin hydrate to determine the effect of CFTR channel activation on bacterial phagocytosis [50,51]. We found that both quercetin and rutin hydrate treatment is able to improve CSE impaired bacterial phagocytosis, but it cannot completely rescue or restore basal levels. The improvement in CSE impaired bacterial phagocytosis by quercetin (~1.49 fold increase) or rutin hydrate (~1.44 fold increase) treatment suggests that either flavonoid mediated increase in CFTR activity or flavonoids directly can modulate phagocytic machinery. Contrary to our expectation, flavonoid treatment was unable to limit bacterial load in media (Fig. 2E) in spite of significantly improved bacterial phagocytosis suggesting that flavonoid may be promoting bacterial growth as well. Hence we next used CFTR(inh)-172, an inhibitor that specifically targets CFTR ion channel activity. We observed that CFTR ion channel inhibition by CFTR(inh)-172 had no effect on bacterial phagocytosis in RAW264.7 macrophages (Fig. 2A-B). Moreover, the role of CFTR ion channels in lysosome acidification and bactericidal activity in macrophages is controversial [34,42,60,62] and our findings at the very least demonstrate that modulating CFTR mediated chloride transport does not affect bacterial phagocytosis. Instead, we anticipated that CFTR dependent lipid rafts serve as a potential mechanism for bacterial phagocytosis based on recent studies discussed above [6,33,38,55].

Moreover, we and others have demonstrated that CS inhibits CFTR dependent rafts and immune responses in inflammatory cells such as macrophages suggesting this as a potential mechanism for bacterial phagocytosis [6,42,55]. Recent studies have also found that CFTR inhibitory factor (cif) secreted by *P*. *aeruginosa* can decrease CFTR expression similar to CS [39,63,64,65]. Hence, in SHS induced infections, *P*. *aeruginosa* is expected to inhibit its phagocytosis by depleting CFTR dependent rafts. We anticipate that restoration of CFTR dependent lipid-rafts can rescue SHS impaired bacterial phagocytosis to control airway infections. Thus, in this study we first evaluated the efficacy of the therapeutic compound VRT-532, a CFTR corrector/potentiator by increasing its membrane stability. We found that VRT-532 can only slightly increase CFTR expression as it may be inhibiting its endocytosis to increase membrane stability [44,66,67]. Although, this minimal CFTR induction by VRT-532 significantly (p<0.05) decreases NF-κB protein expression and reduces bacterial survival (~1.46 fold) in CSE treated cells (Fig. 3), it was unable to significantly improve bacterial phagocytosis. Our data suggest that further induction of CFTR expression can reduce or eliminate bacterial burden. Hence, we evaluated the role of increased m-/r- CFTR expression in bacterial phagocytosis by WT-CFTR transfection. We found that increased CFTR expression leads to significant (p<0.03) rescue of SHS impaired bacterial phagocytosis, while inhibiting its survival (Fig. 4). However, currently available therapeutic regime is unable to provide significant induction of CFTR expression i.e. required to verify the efficacy of CFTR induction in reducing bacterial load from murine/human lungs. We anticipate that targeting CFTR dependent lipid-rafts in CS-COPD can control chronic infection and inflammation by improving CFTR dependent phagocytosis and pro-inflammatory responses [6,12,68,69].

Recent studies suggested a correlation between CFTR levels and *P*. *aeruginosa* internalization *via* lipid-rafts in epithelial cells [35,38,70]. Macrophages may internalize *P*. *aeruginosa* similarly, thus we evaluated if depleting CFTR from cholesterol dependent membrane microdomains by methyl- β-cyclodextran (CD) [12,35,69,71] impairs *P*. *aeruginosa* phagocytosis. We used CD treatments to selectively deplete CFTR from lipid-rafts [6,12,69,71,72] instead of decreasing overall CFTR expression by small interfering RNA (RNAi) and found that CD can in fact significantly inhibit phagocytosis of *PA01*-GFP although it has no significant effect on survival (Fig. 5). The addition of CSE on CD treated macrophages also further impairs bacterial phagocytosis, which indicates a synergistic effect of CSE on CFTR dependent lipid rafts. This is also supported by recent studies showing the role of CS in inducing acquired CFTR dependent lipid-raft dysfunction in COPD. Moreover, CS treatment is known to deplete the number of CFTR ion channels from the apical membrane by inducing its internalization and insolubility [6,7,33]. Hence, CS (FHS/SHS) exposure can similarly deplete m-/r- CFTR expression and lipid-raft activity by inducing its internalization.

In conclusion, we found that SHS exposure impairs bacterial phagocytosis and improves survival to increase alveolar bacterial burden to initiate chronic infection. We also found that modulating CFTR ion channel activity does not alter bacterial phagocytosis in macrophages, while disruption of CFTR dependent lipid-rafts significantly impairs phagocytosis. In contrast, CFTR expression is able to improve bacterial phagocytosis in CSE treated macrophages to reduce bacterial burden. Hence, we propose that restoration of m-/r- CFTR expression may be a promising therapeutic strategy to combat SHS impaired bacterial phagocytosis to control chronic infection and inflammation in COPD-emphysema subjects.
